# The ATCC Genome Portal: Microbial Genome Reference Standards with Data Provenance

**DOI:** 10.1128/MRA.00818-21

**Published:** 2021-11-24

**Authors:** Briana Benton, Stephen King, Samuel R. Greenfield, Nikhita Puthuveetil, Amy L. Reese, James Duncan, Robert Marlow, Corina Tabron, Amanda E. Pierola, David A. Yarmosh, Patrick Ford Combs, Marco A. Riojas, John Bagnoli, Jonathan L. Jacobs

**Affiliations:** a American Type Culture Collection, Manassas, Virginia, USA; University of Southern California

## Abstract

Lack of data provenance negatively impacts scientific reproducibility and the reliability of genomic data. The ATCC Genome Portal (https://genomes.atcc.org) addresses this by providing data provenance information for microbial whole-genome assemblies originating from authenticated biological materials. To date, we have sequenced 1,579 complete genomes, including 466 type strains and 1,156 novel genomes.

## ANNOUNCEMENT

The data provenance (origins) of genome assemblies found in International Nucleotide Sequence Database Collaboration (INSDC) databases increasingly have gaps, errors, and omissions, which collectively create real-world challenges for interpretation and scientific reproducibility relying on genomic data (1–7). Although depositor assurances and INSDC quality pipelines create some trust regarding these data, these approaches fall short of providing complete data provenance information and authentication, since there is no guarantee that the physical biomaterials are available or that the associated metadata are accurate. Further complications include gaps in the recorded chain of custody of the source materials, undisclosed phenotypic differences, variability in naming conventions, and lack of standardized data formats, all of which impact the data provenance and the reliability of public genomic data.

To address the issue of provenance and attribution for genome references, the American Type Culture Collection (ATCC) is systematically sequencing, assembling, and annotating its entire microbial collection. The goal is to provide the research community with provenance information and authentication between the biological source materials and reference genome assemblies derived from them. To date (18 October 2021), 1,579 genome assemblies are available for research use via the ATCC Genome Portal (AGP) (https://genomes.atcc.org), a publicly accessible web portal and genome database. This total includes 1,396 bacterial, 74 fungal, and 109 viral genome assemblies, of which 73% (1,156 genome assemblies) represent genome assemblies of novel organisms and strains.

All genome assemblies in the AGP are derived from authenticated materials available from the ATCC and are part of the ATCC Enhanced Authentication Initiative. In general, this includes one or more of the following: assessment of colony morphology, Gram staining, metabolic profiling, antibiotic susceptibility testing, biochemical reactivity testing, 16S rRNA sequencing, matrix-assisted laser desorption ionization–time of flight mass spectrometry (MALDI-TOF MS), and whole-genome next-generation sequencing.

Bacteria and fungi included in the AGP (1,475 genome assemblies [∼93%]) are sequenced on both Illumina and Oxford Nanopore Technologies (ONT) sequencing platforms, whereas viruses are sequenced only on Illumina systems. All reads are quality filtered, trimmed, and down-sampled to an estimated 100× maximum coverage using MASH (8). ONT reads are filtered and trimmed using Filtlong, whereas fastp is used for Illumina reads (9, 10). Viral and bacterial genomes are assembled *de novo* using SPAdes, and fungal genomes are assembled using MaSuRCA with Flye (11–13). Postassembly quality control and annotation of each genome are performed using CheckM (bacteria), Prokka (bacteria), BUSCO (fungi), and scripts from One Codex (viruses) (14–17).

The AGP represents one of the first microbial genome databases to provide genomic data provenance information between the source materials and their genome assemblies. Our goal is to continue our practice of updating the AGP monthly with new assemblies. In addition, in the near future we will begin to include phenotypic, cell imaging, and lot-specific bioproduction data, as well as additional omic data as they become available.

### Data availability.

The details of specific laboratory methods, DNA extraction and sequencing quality thresholds, bioinformatic pipelines, software parameters, the REST application programming interface (API), bulk downloads of raw data, and the databases used are available online (https://github.com/ATCC-Bioinformatics/AGP-Resource-Announcement).
